# Electrophysiological examination of response-related interference while dual-tasking: is it motoric or attentional?

**DOI:** 10.1007/s00426-019-01261-8

**Published:** 2020-02-04

**Authors:** Kyung Hun Jung, Tim Martin, Eric Ruthruff

**Affiliations:** 1 Department of Psychological Science, Kennesaw State University, 1000 Chastain Road, Kennesaw, GA 30144 USA; 2 Department of Psychology, University of New Mexico, Albuquerque, USA

## Abstract

The possibility that interference between motor responses contributes to dual-task costs has long been neglected, yet is supported by several recent studies. There are two competing hypotheses regarding this response-related interference. *The motor*-*bottleneck hypothesis* asserts that the motor stage of Task 1 triggers a refractory period that delays the motor stage of Task 2. The *response*-*monitoring hypothesis* asserts that monitoring of the Task-1 motor response delays the response-selection stage of Task 2. Both hypotheses predict lengthening of Task-2 response time (RT2) when Task 1 requires motor processing relative to when it does not. However, they assume different loci for the response-related bottleneck, and therefore make different predictions regarding (a) the interaction between Task-1 motor requirement and the Task-2 difficulty effect as measured by RT2 and (b) the premotoric durations and motoric durations of Task 2 as measured by lateralized readiness potentials (LRPs). To test these predictions, we conducted two experiments manipulating the Task-1 motor requirement (Go vs. NoGo) and Task-2 response-selection difficulty, as well as the stimulus-onset asynchrony (SOA). Task-1 motor processing significantly lengthened RT2, suggesting response-related interference. Importantly, the Task-1 motor response reduced the Task-2 difficulty effect at the short SOA, indicating postponement of the Task-2 motor stage, consistent with the motor-bottleneck hypothesis. Further consistent with the motor-bottleneck hypothesis, the Task-2 LRP indicated a consistent premotoric duration of Task 2 regardless of Task-1 motor requirement. These results are difficult to reconcile with the response-monitoring hypotheses, which places the response-related bottleneck before the response-selection stage of Task 2. The results also have important implications regarding use of locus-of-slack logic in PRP studies.

## Introduction

Research on divided attention shows both efficiency and limitation. While visual perception demonstrates successful divided attention under desirable conditions (Shiffrin & Schneider, 1977), dual-task performance is often subject to stubborn limitations. These limitations arise even when the component tasks are simple (Vince, 1948) and sometimes even after participants receive extensive practice (Ruthruff, Johnston, & Van Selst, 2001; Strobach & Schubert, 2017). The overall goal of the present study is to better understand the nature of dual-task interference. In particular, we aim to elucidate the contribution of the motor stage, which traditionally has been neglected as a cause of dual-task interference.

To study the mechanisms of dual-task interference, researchers often use the overlapping-task paradigm, which is also known as the psychological refractory period (PRP) paradigm. In this paradigm, participants make a separate response to each stimulus of two tasks (S1 and S2 for Task 1 and Task 2). Researchers manipulate the degree of temporal overlap between the two tasks by varying the interval between the onsets of S1 and S2 (stimulus-onset asynchrony, SOA). A common finding is that the Task-2 performance is impaired as task overlap increases (i.e., as SOA decreases), which is known as the PRP effect (Telford, 1931; Vince, 1948; Welford, 1952).

The PRP effect implies that, at short SOAs, at least one of the three basic stages of Task 2 (perception, response selection, and motor) is slowed or delayed relative to long SOAs. Thus far, the dominant explanation of the PRP effect has been the response-selection bottleneck hypothesis (Pashler, 1984, 1994; Welford, 1952, but see also Tombu & Jolicœur, 2003, for the capacity-sharing hypothesis), which assumes that only one response-selection stage can access limited-capacity central attentional resources at a time. According to this hypothesis, while the response-selection stage of Task 1 is accessing the limited resources, Task-2 response selection cannot operate and must wait until the resources become available. This waiting period (sometimes called cognitive slack) accounts for the PRP effect.

**Fig. 1:**
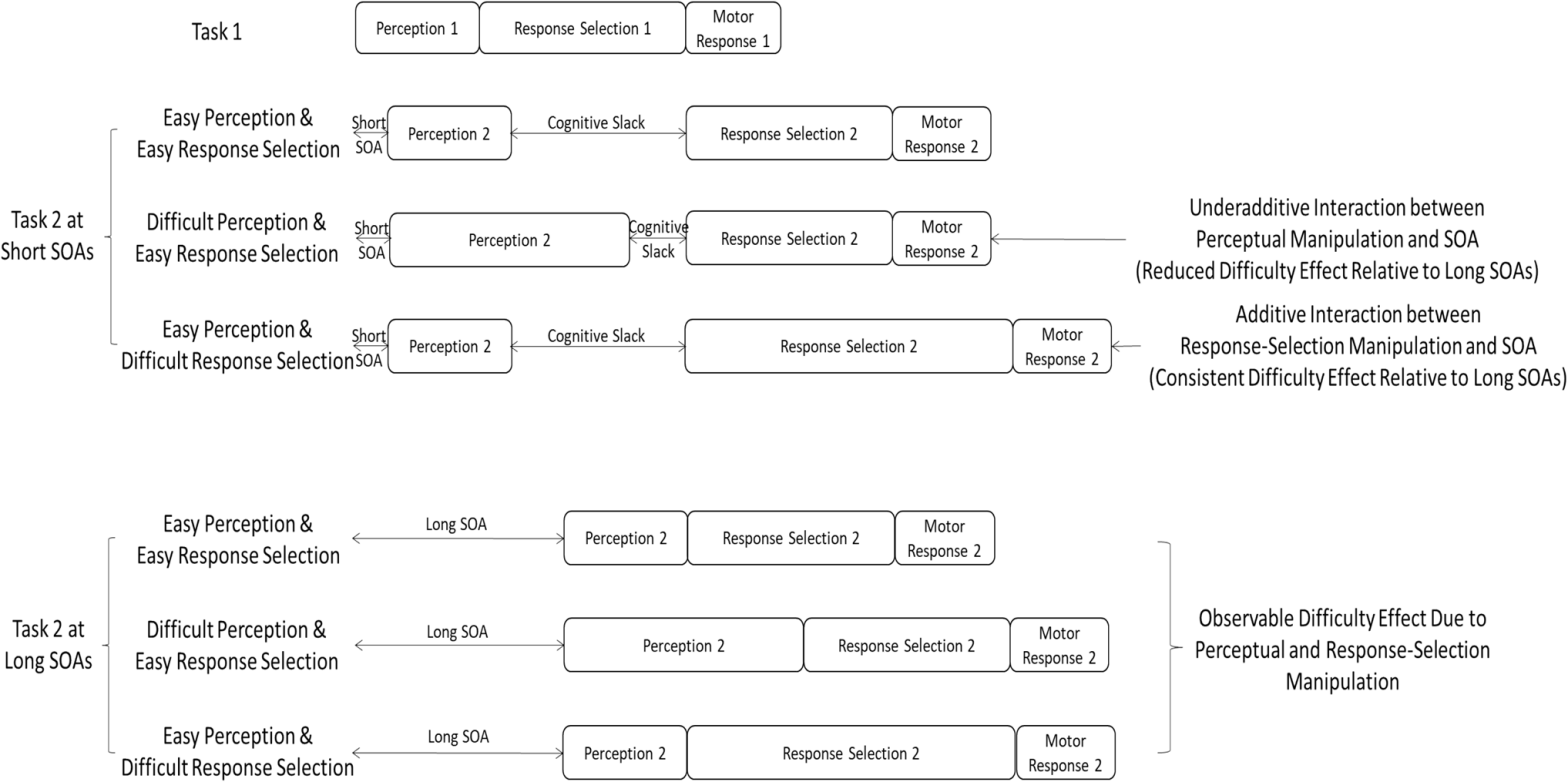
Cognitive slack hypothesized by response selection bottleneck (RSB) hypothesis. According to RSB hypothesis, A2 manipulation would not be reflected in RT2 (upper panel) while B2 manipulation would (lower panel)

Previous studies have supported the core assumption of the response-selection bottleneck hypothesis—that the major dual-task bottleneck is located at the response-selection stage. For example, using “locus-of-slack” logic (McCann & Johnston, 1992), Pashler and Johnston (1989) demonstrated that a manipulation of the perceptual difficulty of Task 2 (dim vs. bright S2) caused the expected difficulty effect on response time of Task 2 (RT2) at long SOAs but not at short SOAs (i.e., an underadditive interaction, see Fig. 1). In contrast, a manipulation of the response-selection difficulty of Task 2 (repeated vs. non-repeated responses) showed a consistent difficulty effect on RT2 across SOAs (i.e., an additive interaction). This pattern of interactions is exactly what the response-selection bottleneck hypothesis predicts. Specifically, the effect of manipulations on Task-2 stages prior to the bottleneck (cognitive slack), such as perceptual manipulations, will be absorbed into the period of cognitive slack at short SOAs but not at long SOAs, yielding an underadditive interaction with SOA. Meanwhile, the effect of manipulations of Task-2 stages after the bottleneck, such as response-selection manipulations, cannot be absorbed into the slack, yielding an additive interaction with SOA.

Critical to the present study, the response-selection bottleneck hypothesis assumes that the motor stage does not participate in the dual-task interference (the capacity-sharing hypothesis has the same assumption). That is, the motor stage of a task can operate in parallel with any stage of the other task. Therefore, according to the response-selection bottleneck hypothesis, manipulating the existence or duration of the motor stage of Task 1 would not necessarily affect RT2. However, several studies (see below) have contradicted this prediction, suggesting that the response-selection hypothesis requires some modification.

## Response-related interference

Response-related interference in dual-task performance was first hypothesized based on an underadditive interaction between Task-2 response-selection difficulty and SOA (in contrast to the additivity observed by Pashler & Johnston, 1989). Such underadditivity suggests that there is a very late bottleneck (i.e., after the response-selection stage), into which the effect of response-selection manipulation can be absorbed. For example, Karlin and Kestenbaum (1968) employed simple-detection vs. two-choice responses to manipulate the response-selection difficulty of Task 2 and found that the effect of this manipulation diminished at short SOAs relative to long SOAs (underadditivity). Based on this finding, Keele (1973) proposed that the initiation of the Task-1 motor response is followed by a motoric refractory period that temporarily prevents initiation of a motor response to Task 2, creating a *response*-*related bottleneck* located *after* the response-selection stage but before the motor stage of Task 2, into which the difficulty effect can be absorbed. Keele further concluded that the primary source of the PRP effect is competition between two motor stages rather than competition between response-selection stages.

De Jong (1993) tested Keele’s (1973) proposal by directly manipulating the need for a motor response to Task 1; he used the go–no-go Task 1, which did not require a motor response on half of the trials. He manipulated the response-selection difficulty of Task 2 using the simple-detection vs. two-choice responses, following Karlin and Kestenbaum (1968). The results supported Keele’s claim of response-related interference by showing underadditivity between Task-2 response-selection difficulty and SOA, which was stronger when Task 1 required a motor response (Go trials) than when it did not (NoGo trials). Therefore, De Jong concluded that the Task-1 motor response imposes a bottleneck (in addition to, but separate from, the response-selection bottleneck) between the response-selection and motor stage of Task 2, into which the effect of response-selection difficulty was absorbed.

The underadditive interaction between Task-2 response-selection difficulty and SOA found in Karlin and Kestenbaum (1968) and De Jong (1993) directly contradicts the additive interaction found in Pashler and Johnston (1989). To explain this inconsistency, later researchers pointed to a possible flaw in the way that Karlin and Kestenbaum, as well as De Jong, manipulated Task-2 response-selection difficulty (i.e., simple-detection vs. two-choice responses). For example, Schubert (1999) pointed out that when participants perform a simple-detection task and the SOA is long, they can anticipate the upcoming stimulus and become increasingly prepared to make the predetermined response while waiting for the stimulus. Therefore, the easy condition (simple detection) at long SOAs is even easier than it is at short SOAs. Because such a preparation is not possible in the difficult condition (two-choice responses), the net result would be an artifactual underadditivity between the response-selection difficulty and SOA. In the present study, therefore, we avoided this specific procedure to manipulate Task-2 response-selection difficulty.

Although the above studies comparing simple-detection with two-choice responses were not definitive, other behavioral and physiological evidence support the response-related interference. For example, Ulrich et al. (2006) manipulated the *duration* of the Task-1 motor response using a response lever that moved along either a short or long track. They found that RT2 was significantly affected by Task-1 motor-response duration: as the duration increased (tracing a long track relative to a short one), RT2 further increased (from 653 to 736 ms) and this increase was even greater at short SOAs than at long SOAs. The results supported the response-related interference assuming that the long-duration motor responses of Task 1 caused greater response-related interference than the short-duration ones especially at short SOAs where Task-2 motor response follows the Task-1 motor response with a close temporal proximity.

As mentioned above, response-related interference would be most prominent at short SOAs—where the two motor responses are due at nearly the same time—than at long SOAs, which can be examined using lateralized readiness potentials (LRP; Coles, 1989). The LRP is an evoked response potential generated by primary motor cortex M1 with likely contributing sources from other motor cortical areas (Praamstra, Schmitz, Freund, & Schnitzler, 1999; Sammer et al., 2005). When a participant is preparing to initiate a motor response, M1 in the contralateral hemisphere of the intended hand generates greater currents than M1 in the ipsilateral hemisphere. This discrepancy in the motor cortex activity between the two hemispheres is the source of the LRP. The LRP is believed to occur as early as during the response-selection stage but before the actual motor response, and therefore, can serve as an index of when the motor response is prepared and initiated (Coles, 1989; Miller & Ulrich, 1998, Miller & Hackley, 1992). If the LRP is calculated relative to the stimulus onset (S-LRP), it reflects the duration of premotoric stages (perception and response selection, Osman & Moore, 1993). If the LRP is calculated relative to the response onset (LRP-R), then it reflects the duration of the motor stage. Importantly, while the response-selection bottleneck hypothesis predicts a lengthening of the premotoric stages of Task 2 only (as measured by S2-LRP) as SOA decreases, if the duration of the motor stage (as measured by LRP-R2) increases as SOA decreases, then, it would be more compatible with the notion of response-related interference. Sommer, Leuthold, and Schubert (2001) replicated Karlin and Kestenbaum (1968)—with the manipulation of whether Task 2 required simple-detection or two-choice responses—while measuring LRPs. In the simple-detection condition, they observed LRP evidence that participants did in fact initiate the response early, in anticipation of the stimulus, consistent with Schubert’s (1999) anticipation account. Therefore, they concluded that the earlier evidence of response-related bottleneck that involved a simple-detection condition was due to the increased anticipation of S2 in the simple-detection condition at long SOAs rather than the response-related bottleneck itself. However, in the two-choice-response condition, which is the typical experimental setting of the PRP paradigm, they found that as SOA decreased (from 700 to 100 ms), both the premotoric duration as measured by the S-LRP and the motor-response duration as measured by the LRP-R of Task 2 increased: 245-ms increase in the premotoric and 55-ms increase in the motoric duration. Such an increase of motoric duration of Task 2 at short SOAs relative to long SOAs is consistent with the response-related interference.

In sum, the notion of response-related interference was originally proposed based on the underadditive interaction between Task-2 response-selection difficulty and SOA, which was later criticized as a possible artifact of using simple detection. However, response-related interference has also been supported by an effect of the Task-1 motor-stage duration on RT2 (Ulrich et al., 2006) as well as by lengthening of the Task-2 motor-stage duration at short SOAs as measured by LRPs (Sommer et al., 2001)—in both studies, the effect of response-related interference was less than 100 ms. Below, we address two competing hypotheses regarding the nature of the response-related interference. Although both hypotheses assert that Task-1 motor stage causes a processing bottleneck in Task 2, they assume the bottleneck in different locations of Task 2.

### Two hypotheses regarding the nature of the response-related interference

Regarding the nature and locus of the response-related interference, there are two prominent competing views: the motor-bottleneck hypothesis and the response-monitoring hypothesis. The *motor*-*bottleneck hypothesis* suggests that the nature of response-related interference is purely motoric. Specifically, it assumes that the initiation of the Task-1 motor response is followed by a motoric refractory period that temporarily prevents the initiation of the Task-2 motor response (Keele, 1973). Therefore, according to this hypothesis, the Task-1 motor response generates an additional slack *after* the response-selection but before the motor stage of Task 2. A competing view, the *response*-*monitoring hypothesis*, suggests that the nature of the response-related interference is attentional rather than motoric. This hypothesis is based on Welford’s (1952) assumption that a motor response is accompanied by a monitoring process that consumes the same capacity-limited central attentional resources as response selection. Therefore, according to the response monitoring hypothesis, Task-2 response selection does not merely wait for completion of Task-1 response selection but also for completion of the Task-1 response and the monitoring of that response. In other words, Task-1 response monitoring adds a period of slack *before* the response-selection stage of Task 2, in a sense extending the cognitive slack already created by the response-selection bottleneck.

**Fig. 2:**
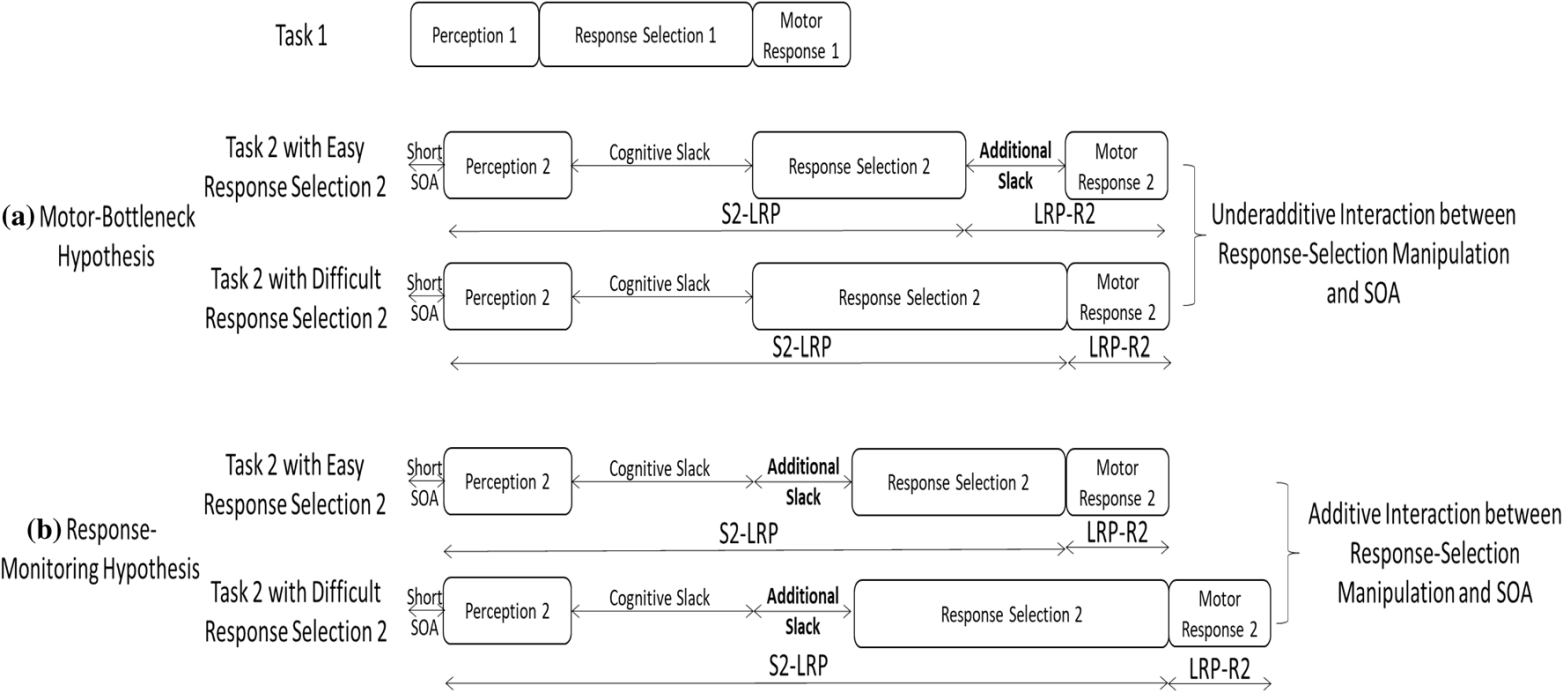
Example processing time diagrams illustrating the distinct predictions of the motor-bottleneck and response-monitoring hypotheses (i.e., where the additional response-related bottleneck occurs). **a** The motor-bottleneck hypothesis assumes that the Task-2 motor stage is delayed by the Task-1 motor stage, resulting in a response-related bottleneck (denoted as ‘additional slack’) located *after* Task-2 response selection. **b** Meanwhile, the response-monitoring hypothesis assumes that Task-2 response selection is delayed while monitoring the Task-1 responses, resulting in a response-related bottleneck *before* the response-selection stage of Task 2. Therefore, the motor-bottleneck hypothesis allows underadditivity between Task-2 response-selection difficulty and stimulus-onset asynchrony (SOA) due to absorption of Task-2 difficulty effect into the additional slack, but the response-monitoring hypotheses does not. Regarding the LRP, the motor-bottleneck hypothesis assumes that the Task-1 motor response lengthens the duration of Task 2 *after* the response-selection stage (because the additional slack is inserted after the response selection of Task 2), and this effect is especially prominent in the easy condition of Task 2. The response-monitoring hypothesis, however, assumes that Task-1 motor response lengthens the duration of Task 2 prior to response selection (i.e., the additional slack is inserted *before* Task-2 response selection)

Before describing evidence supporting each of the two hypotheses, we should emphasize the critical distinction between the two hypotheses: whether the additional processing bottleneck in Task 2 triggered by the Task-1 motor response is located *before or after* the response-selection stage of Task 2, which could affect RT2 and Task-2 LRP differentially. Regarding RT2, because the motor-bottleneck hypothesis assumes the response-related bottleneck *after* the response-selection stage of Task 2 (noted as additional slack in a in Fig. 2), it allows absorption of any Task-2 response-selection-difficulty effects into the slack, resulting in an *underadditive* interaction between response-selection difficulty and SOA. In contrast, because the response-monitoring hypothesis assumes the additional slack *before* the response-selection stage of Task 2 (b in Fig. 2), the effect of Task-2 response-selection difficulty cannot be absorbed into the slack, resulting in an *additive* interaction between response-selection difficulty and SOA on RT2. Regarding the LRP, the motor-bottleneck hypothesis suggests that the Task-1 motor response lengthens the duration of Task 2 *after* the response-selection stage (because the additional slack is inserted after the response selection of Task 2); in contrast, the response-monitoring hypothesis suggests that the Task-1 motor response lengthens the duration before response selection (because the additional slack is inserted *before* the Task-2 response selection).

#### Evidence for the motor-bottleneck hypothesis

Bratzke, Rolke, and Ulrich (2009) manipulated the duration of the Task-1 motor response (tracing a short or long track) as well as the difficulty of Task-2 response selection (compatible vs. non-compatible stimulus–response mapping). They found that the Task-2 difficulty effect was smaller at short SOAs than at long SOAs—121 ms vs. 165 ms (underadditivity)—and this underadditivity was more prominent when the Task-1 motor-response duration was long (67-ms interaction) than when it was short (22-ms interaction). This is just the result one would expect from the motor-bottleneck hypothesis: lengthening of the Task-1 motor response further lengthens the additional slack period before the Task-2 motor response, into which the effect of Task-2 response-selection difficulty can be absorbed. Meanwhile, the response-monitoring hypothesis, which assumes additional slack before the response-selection stage of Task 2, incorrectly predicts an additive interaction between Task-2 response-selection difficulty and SOA.

#### Evidence for the response-monitoring hypothesis

Evidence supporting the response-monitoring hypothesis involves what is known as the residual PRP effect: even without any temporal overlap between the processes of Task 1 and Task 2—because S2 is presented *after* the completion of Task 1—RT2 is still prolonged at short SOAs relative to long SOAs. As an example of such a residual PRP effect, in one condition of Jentzsch, Leuthold, and Ulrich (2007), mean RT2 was 40 ms longer at the short SOA (400 ms) than the long SOA (600 ms) even though S2 was presented after the completion of Task 1.

To evaluate the predictions of the motor-bottleneck and response-monitoring hypotheses regarding the residual PRP effect, Jentzsch et al. (2007) analyzed the LRP and found that the premotoric duration of Task 2 (S2-LRP) increased as SOA decreased, while the motor-response duration (LRP-R2) was consistent across SOAs (unlike the findings of Sommer et al., 2001). This result, along with their behavioral data, is more compatible with the response-monitoring hypothesis that assumes the additional slack due to Task-1 motor response *before* the Task-2 response selection than the motor-bottleneck hypothesis.

In sum, behavioral measures (RT2) obtained with a Task-1 motor-duration manipulation supported the motor-bottleneck hypothesis by showing prominent underadditivity between Task-2 response-selection difficulty and SOA especially with a long motor stage of Task 1 (Bratzke et al., 2009). In contrast, the LRP data regarding the residual PRP effect supported the response-monitoring hypothesis by showing a lengthened premotoric duration of Task 2 at short SOAs along with a consistent motor-stage duration across SOAs (Jentzsch et al., 2007; but see also, Sommer et al., 2001). We should note that the evidence supporting each hypothesis was obtained with different contextual factors: (a) whether the researchers focused on the traditional PRP effect or the residual PRP effect, (b) whether the key dependent measures were behavioral (RT2, Bratzke et al., 2009) or include electrophysiological measures (LRP, Jentzsch et al., 2007), and (c) whether the primary cause of the response-related interference (i.e., Task-1 motor response) was manipulated: Bratzke et al. (2009) manipulated the duration of Task-1 motor response while Jentzsch et al. (2007) did not. To help resolve the debate regarding the two hypotheses while addressing these methodological discrepancies, we directly manipulated the Task-1 motor requirement, focused on the traditional PRP effect (rather than the residual PRP effect), while obtaining both behavioral and electrophysiological measures at the same time.

## The present study

The purpose of the present study is to test the two competing hypotheses regarding the nature of response-related interference. We manipulated the following two factors that should differentially affect Task 2 in terms of both RT2 and LRP according to the two hypotheses: (a) the primary cause of the response-related interference: whether Task 1 requires a motor response (Go vs. NoGo) and (b) Task-2 response-selection difficulty (compatible vs. non-compatible stimulus–response mapping).

At long SOAs (900 ms in Experiment 1 and 1200 ms in Experiment 2), which have minimal response-related interference, both hypotheses assume that Task 2 is performed with little influence from the Task-1 motor requirement. Therefore, at long SOAs, there should be an observable Task-2 difficulty effect on RT2 and LRPs regardless of Task-1 motor requirement. That is, RT2 and S2-LRP (the premotoric duration) should be longer in the difficult condition than in the easy condition, while the LRP-R2 (the motoric duration) should be independent of Task-2 difficulty as well as Task-1 motor requirement.

**Fig. 3:**
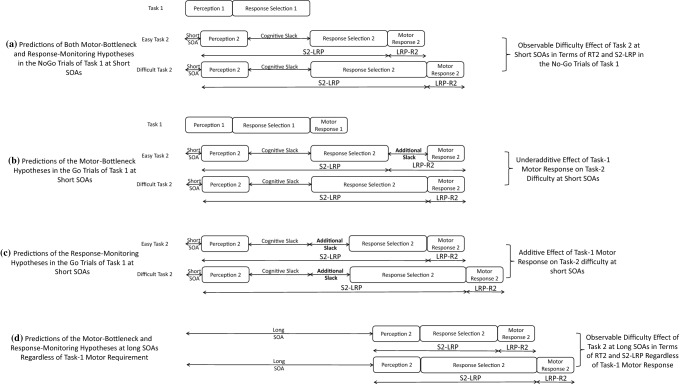
Predictions of the motor-bottleneck and response-monitoring hypotheses as a function of Task-1 motor requirement, Task-2 difficulty, and stimulus-onset asynchrony (SOA). According to the motor-bottleneck hypothesis, the response-related bottleneck (causing the additional slack) that occurs *after* the response selection of Task 2 can absorb the Task-2 difficulty effect in the Go trials but not in the NoGo trials at short SOAs (compare **a**, **b**, and **d**). In contrast, the response-monitoring hypothesis assumes that the additional slack is located *before* the response selection of Task 2 and cannot absorb Task-2 difficulty effect regardless of Task-1 motor requirement (panel **c**). Regarding the premotoric and motoric durations of Task 2, the motor-bottleneck hypothesis predicts a consistent premotoric duration (S2-LRP) regardless of Task-1 motor requirement, along with a lengthened motoric duration (LRP-R2) due to Task-1 motor response only in the *easy* condition of Task 2 at short SOAs (**a**, **b**). Meanwhile, the response-monitoring hypothesis predicts a lengthened premotoric duration of Task 2 in the Go trials compared to the NoGo trials at short SOAs along with a consistent motoric duration (**a**, **c**)

**Fig. 4:**
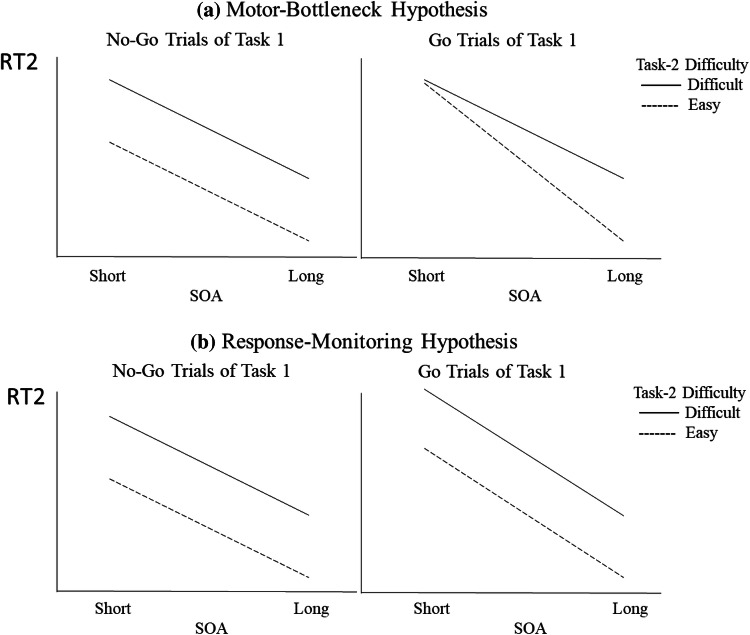
Predictions of the motor-bottleneck and response-monitoring hypotheses on Task-2 response time (RT2) as a function of Task-1 motor requirement, Task-2 difficulty, and stimulus-onset asynchrony (SOA). **a** According to the motor-bottleneck hypothesis, at short SOAs, Task-2 difficulty effect should be reduced more strongly in the Go trials of Task 1 than in the NoGo trials. **b** According to the response-monitoring hypothesis, at short SOAs, RT2 should be increased in the Go trials of Task 1 relative to the NoGo trials, and this increase should be similar for the easy and difficult conditions

At the short SOA, which has maximal response-related interference, the two hypotheses predict differential effects of Task-1 motor requirement on RT2 and LRPs. Regarding RT2, the most distinguishable prediction between the two hypotheses is, as discussed earlier, whether the Task-2 difficulty effect is reduced when Task 1 requires a motor response. According to the motor-bottleneck hypothesis, Task-1 motor processing creates the response-related bottleneck after the response-selection stage of Task 2 (b in Fig. 3). Therefore, it predicts absorption of the Task-2 difficulty effect into the slack when Task 1 requires a motor response (Go trials) but not when Task 1 does not require a response (NoGo trials), resulting in a two-way interaction between Task-1 motor requirement and Task-2 difficulty on RT2 (compare a and b in Fig. 3). In contrast, the response-monitoring hypothesis asserts that Task-1 motor response generates the response-related bottleneck before the response-selection stage of Task 2 (c in Fig. 3). Therefore, it predicts a consistent lengthening of RT2 due to Task-1 motor response in both the easy and difficult conditions of Task 2—no interaction between Task-1 motor requirement and Task-2 difficulty (compare a and c in Fig. 3). Figure 4 shows the predictions of the two hypotheses on RT2.

In addition, the motor-bottleneck hypothesis predicts a three-way interaction between Task-1 motor requirement, Task-2 difficulty, and SOA: the two-way interaction between Task-1 motor requirement and Task-2 difficulty will be observed at the short SOA but not at the long SOA. In contrast, the response-monitoring hypothesis does not predict such a three-way interaction.

The two competing hypotheses also make different predictions regarding the premotoric duration of Task 2 (as measured by the S2-LRP) at the short SOA. Because the motor-bottleneck hypothesis assumes that the Task-1 motor response inserts the additional slack after the response selection of Task 2, the hypothesis predicts a consistent premotoric duration of Task 2 regardless of Task-1 motor requirement (i.e., compare a and b in Fig. 3). In contrast, because the response-monitoring hypothesis assumes that the response-related bottleneck occurs before the response-selection stage of Task 2, Task-1 motor response lengthens the premotoric durations of Task 2 regardless of Task-2 difficulty (compare a and c). Neither hypothesis predicts a significant three-way interaction on S2-LRP across the three factors because they both assume consistent Task-2 difficulty effect across conditions.

Finally, regarding the motoric duration of Task 2 (as measured by LRP-R2), both hypotheses predict that it should be invariant across conditions, with just one exception: the motor-bottleneck hypothesis predicts a lengthening of LRP-R2 when Task 2 is easy, the SOA is short, and Task 1 requires a motor response. This exception results from the motor-bottleneck hypothesis’ assumption that, in this specific condition, the response-selection stage of Task 2, during which the LRP begins, is first followed by the motor refractory period and then followed by the motor response (see the easy condition of Task 2 in b in Fig. 3). Table 1 summarizes the predictions of the two hypotheses with respect to RT2, S2-LRP, and LPR-R2.

**Table 1:** Summary of predictions of the motor-bottleneck hypothesis and the response-monitoring hypothesis for the short stimulus-onset asynchrony (SOA) with respect to Task-2 mean response time (RT2), S2-LRP, and LRP-R2

Hypotheses and results	Predictions		
	RT2	S2-LRP	LRP-R2
The motor-bottleneck hypothesis	Reduced Task-2 difficulty effect when Task-1 requires a motor response (Go trials) relative to when it does not (NoGo trials)	Consistent effect of Task-2 difficulty regardless of Task-1 motor requirement	LRP-R2 in the easy condition of Task 2 is longer when Task-1 requires a response (Go trials) than when it does not (NoGo Trials)
The response-monitoring hypothesis	Consistent Task-2 difficulty effect regardless of Task-1 motor requirement	Main effect of Task-1 motor requirement (longer S2-LRP by Task-1 motor response)	Consistent LRP-R2

## Experiment 1

### Method

#### Participants

Twenty-seven undergraduate students from Kennesaw State University and the first two authors of this paper participated in Experiment 1. For the LRP analysis, five participants were excluded due to excessive noise on either electrode C3 or C4 which precluded calculation of the LRP (their behavioral data were included in RT and ACC analysis). Another was lost due to excessive EEG noise on all channels on approximately half of all trials. Another two participants were excluded because of their low accuracy (lower than 80% for either Task 1 or Task 2). Among the remaining participants (ten women, eleven men, *M*_age_ = 23.57, age range = 18–46), seven participants were African American, eleven were White (non-Hispanic), two were Asian, and one reported multiple ethnicities. Three reported being left-handed and all reported normal or corrected-to-normal vision.

#### Stimuli and apparatus

The auditory stimuli of Task 1 (S1) were three sinusoidal tones of 800, 1000, and 1200 Hz. The visual stimuli of Task 2 (S2) were arrows pointing either left, up, right, or down (approximately 2-cm length and 0.6-cm width with the viewing distance of about 70 cm). On each trial, one of the three tones was presented over two speakers located on either side of the monitor in front of the participant and one of the four arrows was presented in the middle of the monitor. Participants pressed the *z*, *x*, > , and ? keys located at the corners of the second-bottom row of a keyboard to respond. They rested the left middle, left index, right index, right middle fingers on those four keys, respectively. The second author programmed the experiment in C using functions of the Allegro open source game library and compiled with MinGW. Stimuli were controlled by a desktop Windows PC.^1^

We recorded the electroencephalogram (EEG) using a 40-channel NuAmps amplifier (Neuroscan Compumedics, Inc.) sampling at 1000 Hz with a Quik-Cap for electrode placement while keeping electrode impedances below 10 kΩ. We processed the signals using EEGLAB 4.51 (Delorme & Makeig, 2004). We downsampled data to 500 Hz and filtered with a 0.1-Hz high-pass and 45-Hz low-pass filter. Data were epoched relative to both stimuli and responses using only trials with correct responses. We removed data for bad channels or epochs of excessive noise based on visual inspection. For eye blink and other artifacts, we used an independent component analysis approach (Delorme & Makeig, 2004). After removing the artifacts, we reconstructed the EEG. To calculate the LRP, event-related potential (ERP) data were further filtered with a 4-Hz low-pass. Stimulus-locked averages were taken from 500 ms pre-stimulus to 1500 ms post-stimulus with the pre-stimulus epoch serving as a baseline. Response-locked averages were taken from 1000 ms before to 1000 ms after the response, with a 100-ms baseline from 1000 to 900 ms before the response. Averages were calculated separately for each condition and side of response. The LRP was then calculated according to Coles’s (1989) formula: LRP = [C3 − C4 (right response) + C4 − C3 (left response)]/2. The S-LRP peak was then defined as the minimum in the epoch 500 ms prior to the mean response time, while the LRP-R peak was defined as the minimum in the 500 ms prior to the response. To estimate LRP onset, we used the jackknife procedure of Miller, Patterson, and Ulrich (1998) instead of the segmented regression approaches (Mordkoff & Gianaros, 2000; Schwarzenau, Falkenstein, Hoormann, & Hohnsbein, 1998) because the jackknife procedure is particularly robust to low signal–noise ratio (Ulrich & Miller, 2001). In this procedure, several LRP waveforms were calculated, each time leaving out a single subject’s waveform. To estimate LRP onset, the peak of the LRP was first identified. For the S-LRP, this was defined as the minimum amplitude (i.e., greatest negative deviation) in the 500 ms preceding the average RT for that condition. For the LRP-R, the peak was defined as the minimum in the epoch 500 ms prior to the response. Then, the LRP onset was determined by moving back in time from the peak until the point at which the waveform first reached 50% (S-LRP) or 90% (LRP-R) of the peak amplitude, following the suggestion of Miller et al. After removal of error, grouping, and outlier trials, the least number of trials for any subject in any condition was 27 (in the difficult condition of Task 2, NoGo Task 1, and at the short SOA).

#### Design and procedure

A fixation dot (0.25 cm × 0.25 cm) was presented in the middle of the screen and was present at all times except when error feedback was given or it was covered by S2 (arrow target). At the beginning of each trial, S1 (a tone) was presented for 100 ms. After one of two SOAs (100 or 900 ms), S2 (an arrow) was presented for 500 ms, after which a response period lasted up to 3000 ms or until a response to S2 was emitted. Upon the response, a random inter-trial interval between 1 and 2 s was initiated.

In Task 1, participants were instructed to indicate whether the tone was low (800 Hz) or high (1200 Hz) by pressing the *x* and > keys using the index finger of each hand (Go trials). When S1 was a medium tone (1000 Hz), they were to withhold their response and perform only Task 2 (NoGo trials). In Task 2, participants were instructed to indicate the arrow’s direction by pressing the *z* and ? keys using the middle finger of each hand. For the horizontal arrows, the stimulus–response mapping was compatible (the easy condition of Task 2): the left arrow corresponded to the left middle finger (*z* key) and the right arrow corresponded to the right middle finger (? key). For the vertical arrows, the mapping was non-compatible (the difficult condition of Task 2): the vertical arrows required presses of horizontally aligned keys. The non-compatible mapping was counterbalanced across participants so that some participants were given the up = right/down = left mapping and others the up = left/down = right mapping.

Participants were instructed to respond to both tasks as quickly and accurately as possible with priority placed on Task 1. Feedback was presented immediately after the response to S2. If the Task-1 response was incorrect, the message, for example, “ERROR. REMEMBER X for LOW, > for HIGH! No response for the MIDDLE tone!” was presented for 3 s, with the message tailored to the tone-key mapping. If the Task-2 response was incorrect, the message “ERROR. REMEMBER Z FOR LEFT OR UP ARROW, /KEY FOR RIGHT OR DOWN ARROW” was presented for 3 s, with the message tailored to the mapping between the arrows and keys. If both responses were incorrect on a trial, the Task-1 feedback was given for 3 s, followed immediately by the Task-2 feedback for an additional 3 s. No feedback was given for correct trials.

There was a total of 672 trials (three tones × two SOAs × four arrows × 28 repetition; the order of these trials was completely randomized). Before the main experimental block, participants performed a tone-only practice block of 24 trials, an arrow-only practice block of 24 trials, and dual-task practice block of 48 trials. The experiment lasted approximately 2 h, including preparation, and participants took a break in the middle of the experiment.

## Results

We excluded the practice trials and the first 10 of the experimental trials from data analysis. We excluded trials if RT1 or RT2 was below or above 2.5 standard deviations from that participant’s mean (3% of all trials). We also removed trials (2% of all trials) consistent with response grouping, defined as inter-response-interval ≤ 100 ms (see Ulrich & Miller, 2008). Finally, we removed trials from RT and ERP analysis if the responses were incorrect for either Task 1 or Task 2 (15% of all trials). The resulting mean RTs and accuracies (ACCs) for Task 1 and Task 2 are shown in Table 2.

**Table 2:** Mean response time (RT, in milliseconds) and accuracy (ACC, in percentages) for Task 1 and 2 of Experiment 1 and 2 as a function of Task-1 motor-response requirement (Go vs. NoGo), Task-2 difficulty (easy vs. difficult), and stimulus-onset asynchrony (SOA; short vs. long)

	Task	Task-1 Motor response	Task-2 Difficulty	SOA			
				Short (100 ms)		Long (900 ms in Experiment 1; 1200 ms in Experiment 2)	
				RT (ms)	ACC (%)	RT (ms)	ACC (%)
Experiment 1	Task 1	Go	Easy	1118	85	1199	91
			Difficult	1171	91	1226	93
		NoGo	Easy	–	91	–	93
			Difficult	–	90	–	92
	Task 2	Go	Easy	1342	92	790	98
			Difficult	1527	89	1021	91
		NoGo	Easy	1164	92	692	99
			Difficult	1486	88	996	92
Experiment 2	Task 1	Go	Easy	1007	90	1003	96
			Difficult	1044	96	980	96
		NoGo	Easy	–	94	–	97
			Difficult	–	95	–	97
	Task 2	Go	Easy	1301	93	583	98
			Difficult	1468	92	816	92
		NoGo	Easy	1173	92	561	100
			Difficult	1459	91	852	93

### RT analysis

A repeated-measures analysis of variance (ANOVA) was conducted with the factors of Task-1 motor requirement (Go vs. NoGo), Task-2 difficulty (easy vs. difficult), and SOA (100 ms vs. 900 ms) on RT1 (Go trials only) and RT2. Regarding RT1, there was a main effect of SOA, *F*(1, 25) = 5.863, *p* = 0.023, *η*_*p*_^2^ = 0.190: RT1 was 68 ms longer at the long SOA than at the short SOA. There was also a main effect of Task-2 difficulty on RT1, *F*(1, 25) = 6.763, *p* = 0.015, *η*_*p*_^2^ = 0.213: RT1 was 40 ms longer in the difficult condition of Task 2 than in the easy condition. However, SOA and Task-2 difficulty did not show a significant interaction effect on RT1, *F*(1, 25) = 2.097, *p* = 0.160, *η*_*p*_^2^ = 0.077.

**Fig. 5:**
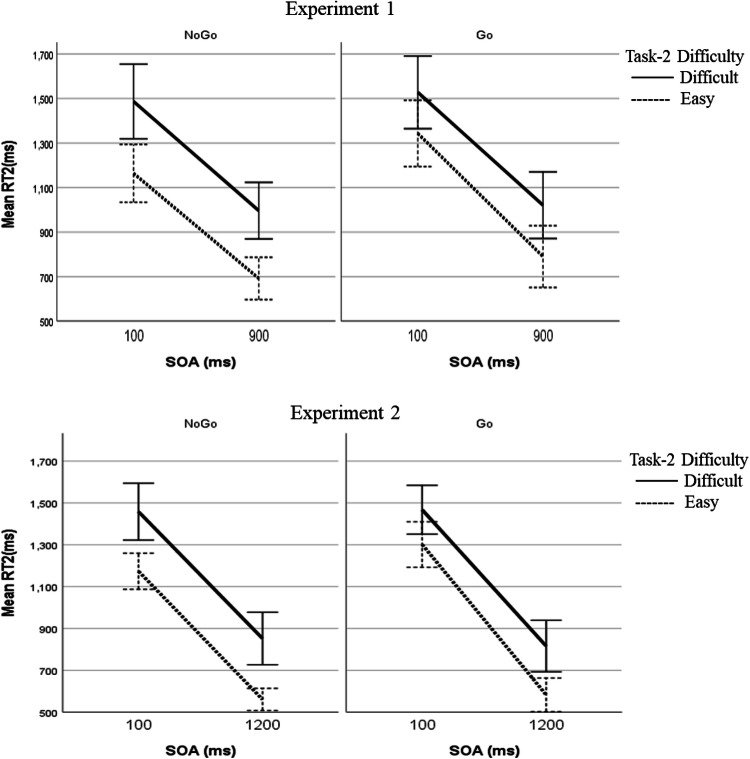
Response time to Task 2 (RT2) as a function of Task-1 motor requirement, Task-2 difficulty, and stimulus-onset asynchrony (SOA) in Experiment 1 and Experiment 2. Error bars show the 95% confidence interval

RT2 was greater at the short SOA (1380 ms) than at the long SOA (875 ms) by 505 ms, showing the typical PRP effect (see the upper panels in Fig. 5), *F*(1, 25) = 527.815, *p* < 0.001, *η*_*p*_^2^ = 0.955. According to the response-selection bottleneck hypothesis (Pashler, 1984, 1994; Welford, 1952), such an increase of RT2 at the short SOA is due entirely to the postponement of the response selection of Task 2 during Task-1 response selection (i.e., the cognitive slack), without any motor-related interference. Contrary to this assumption, the need for a Task-1 motor response lengthened RT2 by 86 ms: RT2 was slower in the Go trials (1170 ms) than in the NoGo trials (1085 ms), *F*(1, 25) = 19.224, *p* < 0.001, *η*_*p*_^2^ = 0.435, supporting the hypothesis of response-related interference. As predicted by both hypotheses being tested, such an effect of Task-1 motor requirement on RT2 was more prominent at the short SOA (110 ms) than at the long SOA (62 ms), *F*(1, 25) = 5.320, *p* = 0.030, *η*_*p*_^2^ = 0.175. Looking at this differently, the overall delay of RT2 at the short SOA relative to the long SOA (the PRP effect) was 481 ms in the NoGo trials while it was 529 ms in the Go trials. That is, when Task 1 requires a motor response, the PRP effect is even greater, suggesting that response-related interference contributes to the PRP effect that typically involves Task-1 motor response.

The data confirmed that our manipulation of Task-2 difficulty was successful: at the long SOA, RT2 was 268 ms greater in the non-compatible stimulus–response condition (difficult condition: 1009 ms) than in the compatible condition (easy condition: 741 ms), *F*(1, 25) = 151.162, *p* < 0.001, *η*_*p*_^2^ = 0.858. As discussed earlier (see also Table 1), the critical test between the motor-bottleneck and the response-monitoring hypotheses concerns whether such a Task-2 difficulty effect is reduced at the short SOA when Task 1 requires a motor response and whether there is a three-way interaction across the three experimental factors (the two-way interaction between Task-1 motor requirement and Task-2 difficulty appears at the short SOA but not at the long SOA). Looking at the short SOA only, the comparison between Go trials and NoGo trials with respect to the Task-2 difficulty effect showed that the difficulty effect was significantly smaller in the Go trials (185 ms) than in the NoGo trials (332 ms), *F*(1, 25) = 39.721, *p* < 0.001, *η*_*p*_^2^ = 0.614. Such a reduction in the Task-2 difficulty effect by the Task-1 motor response indicates that the response-related bottleneck occurs after the response-selection stage of Task 2, consistent with the motor-bottleneck hypothesis; in contrast, the response-monitoring hypothesis does not allow such a reduction because of the assumption that Task-1 motor requirement affects the stages before the response selection of Task 2. Looking at the long SOA, there was another significant interaction between Task-1 motor requirement and Task-2 difficulty, *F*(1, 25) = 12.227, *p* = 0.002, *η*_*p*_^2^ = 0.328: similar to the interaction pattern at the short SOA, the difficulty effect was significantly smaller in the Go trials (231 ms) than in the NoGo trials (304 ms). Although the significant three-way interaction across Task-1 motor requirement, Task-2 difficulty, and SOA indicated that this latter interaction at the long SOA (reduced Task-2 difficulty due to Task-1 motor response) was not as strong as the one at the short SOA, *F*(1, 25) = 4.846, *p* = 0.037, *η*_*p*_^2^ = 0.162, such a modulation of Task-2 difficulty by Task-1 motor requirement even at the long SOA was somewhat unexpected. Both hypotheses predicted a significant difficulty effect at the long SOA regardless of the Task-1 motor requirement. We discussed the possible cause the Task-2 difficulty modulation by Task-1 motor requirement even at the long SOA in the discussion section of this experiment.

### Accuracy (ACC) analysis

Task-1 accuracy (ACC1) was significantly lower at the short SOA than at the long SOA (89% vs. 92%), *F*(1, 25) = 43.938, *p* < 0.001, *η*_*p*_^2^ = 0.637. ACC1 was greater when Task 2 was difficult (92%) than when it was easy (90%), *F*(1, 25) = 11.840, *p* = 0.002, *η*_*p*_^2^ = 0.321. The effect of Task-2 difficulty on ACC1 was greater at the short SOA (− 3%) than at the long SOA (− 1%), *F*(1, 25) = 4.509, *p* = 0.044, *η*_*p*_^2^ = 0.153. The difficulty effect was also greater in the Go trials (− 4%) than in the NoGo trials (1%), *F*(1, 25) = 18.821, *p* < 0.001, *η*_*p*_^2^ = 0.429. There was also a significant three-way interaction across the three experimental factors, indicating that the Task-2 difficulty effect on ACC1 was greater at the short SOA than at the long SOA but only in the Go trials (− 4%), while it was the same across SOAs in the NoGo trials (1%), *F*(1, 25) = 6.532, *p* = 0.017, *η*_*p*_^2^ = 0.207.

Task-2 accuracy (ACC2) was significantly lower at the short SOA than at the long SOA (90% vs. 95%), *F*(1, 25) = 54.060, *p* < 0.001, *η*_*p*_^2^ = 0.684. Task-2 difficulty manipulation showed the expected effect on ACC2: 95% vs. 90% in the compatible and non-compatible conditions, *F*(1, 25) = 28.842, *p* < 0.001, *η*_*p*_^2^ = 0.536. There was a significant interaction between Task-2 difficulty and SOA, *F*(1, 25) = 8.495, *p* = 0.007, *η*_*p*_^2^ = 0.254, suggesting that the difficulty effect was smaller at the short SOA (4%) than at the long SOA (7%). However, Task-1 motor requirement did not show any significant main or interaction effects on ACC2 (*p*s > 0.05).

### LRP analysis

**Fig. 6:**
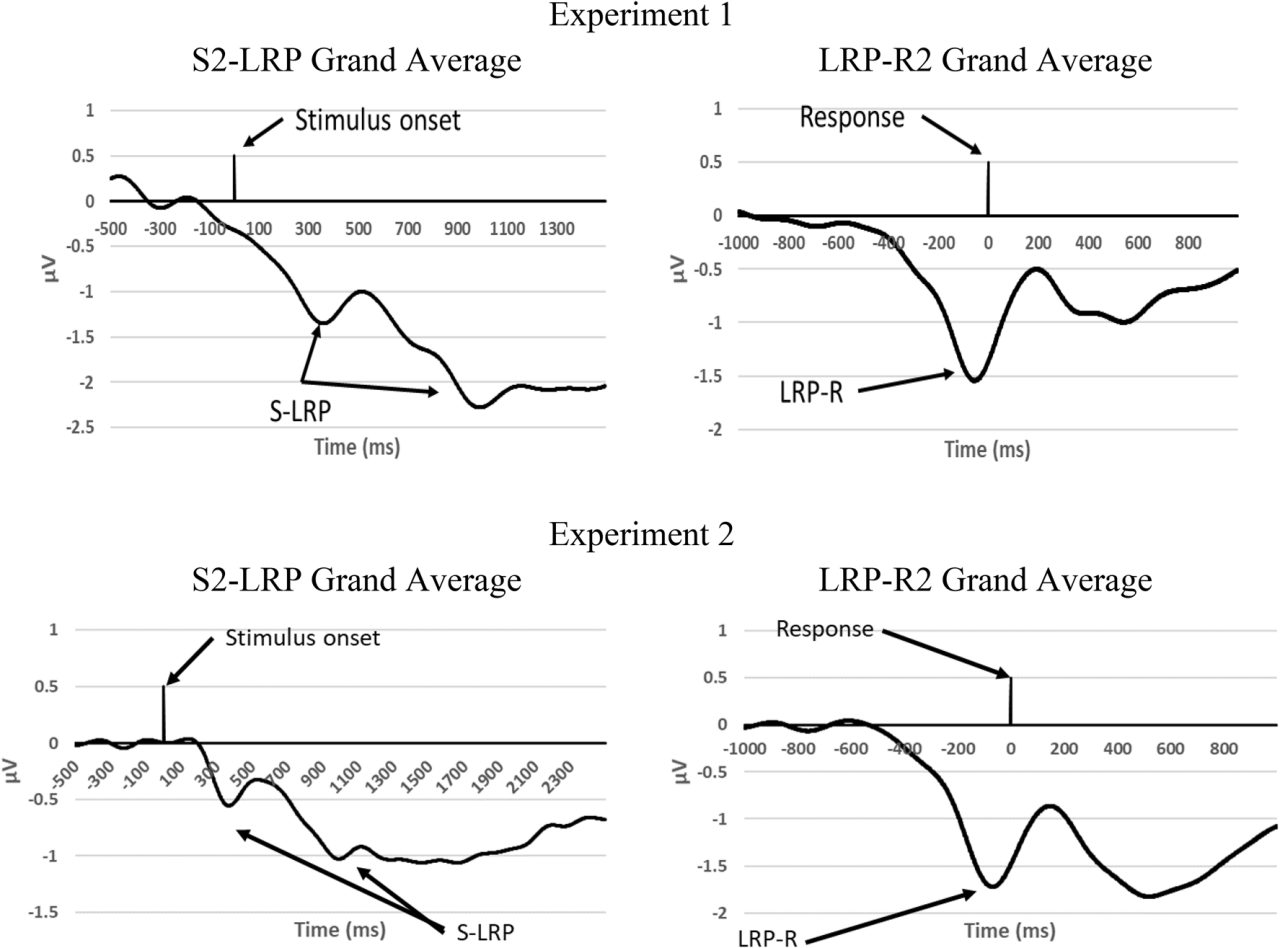
Grand averaged S2-LRP and LRP-R2 of Task 2 in Experiment 1 and Experiment 2. Negative voltages are plotted downward

**Fig. 7:**
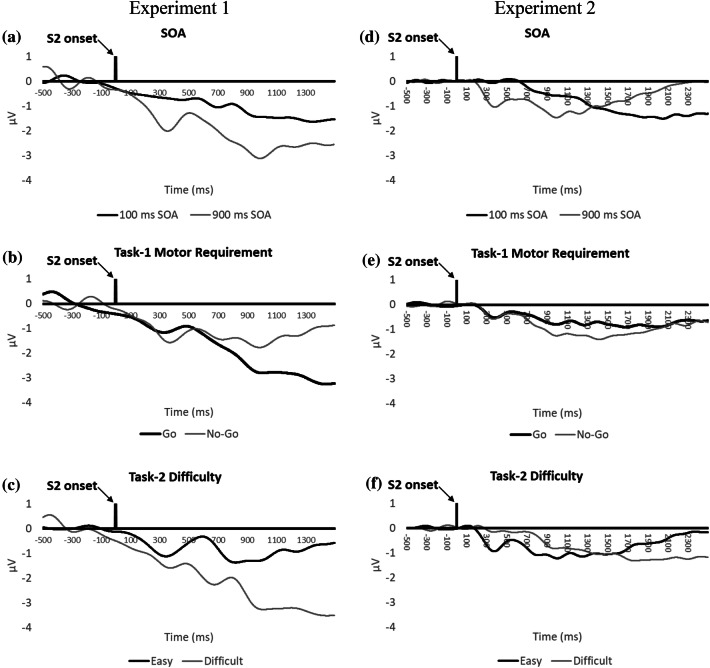
Task-2 S2-LRP in Experiment 1 and Experiment 2. Grand averages are shown as a function of stimulus-onset asynchrony (SOA; **a**, **d**), Task-1 motor requirement (**b**, **e**), and Task-2 difficulty (**c**, **f**)

Grand averaged LRPs for Task 2 are illustrated in Fig. 6. The S2-LRP is shown in Fig. 7 as a function of SOA, Task-1 motor requirement, and Task-2 difficulty. The mean onsets of S2-LRP and LRP-R2 of Task 2 are reported in Table 3.

**Table 3:** Mean onset of S2-LRP and LRP-R2 for Task 2 (in milliseconds) as a function of Task-1 motor-response requirement (Go vs. NoGo), Task-2 difficulty (easy vs. difficult), and stimulus-onset asynchrony (SOA; short vs. long) in Experiment 1 and 2

	Task-1 Motor response	Task-2 Difficulty	SOA			
			Short (100 ms)		Long (900 ms in Experiment 1; 1200 ms in Experiment 2)	
			S2-LRP	LRP-R2	S2-LRP	LRP-R2
Experiment 1	Go	Easy	705	− 96	266	− 104
		Difficult	860	− 114	810	− 82
	NoGo	Easy	564	− 110	255	− 90
		Difficult	1072	− 117	364	− 122
Experiment 2	Go	Easy	910	− 111	230	− 119
		Difficult	1365	− 106	302	− 95
	NoGo	Easy	632	− 165	228	− 134
		Difficult	1168	− 122	706	− 109

Regarding the S2-LRP, the main effect of SOA was significant (*p* = 0.012): the onset of the S2-LRP was delayed at the short SOA (800 ms) relative to the long SOA (424 ms), indicating a lengthening of the premotoric duration of Task 2 as task overlap increases, consistent with the cognitive slack of the response-selection bottleneck hypothesis (Osman & Moore, 1993; Pashler, 1984, 1994). The main effect of task difficulty on S2-LRP was also significant (*p* = 0.022). The onset was delayed in the difficult condition (777 ms) relative to the easy condition (448 ms). No other effects or interactions were significant (*p* > 0.05). Notably, unlike what the response-monitoring hypothesis predicted, there was no significant main effect of Task-1 motor requirement on S2-LRP onset at the short SOA; furthermore, while the response-monitoring hypothesis predicted a consistent *delay* of S2-LRP due to Task-1 motor response at the short SOA, the S2-LRP onset in the difficult condition occurred (at least numerically) *earlier* in the Go trials (860 ms) than in the NoGo trials (1072 ms).

The results did not match one of the predictions of the motor-bottleneck hypothesis. This hypothesis predicted a selective lengthening of LRP-R2 when Task 1 required a motor response for the easy condition of Task 2, at the short SOA. However, none of the effects on LRP-R2 onset were significant (*p*s > 0.05). If anything, LRP-R2 occurred later (− 96 ms) in this specific condition than in other conditions (e.g., − 114 ms in the difficult condition of Task 2, at the short SOA, when Task 1 required a motor response; see Table 3).

### Discussion

Overall, the results better matched the predictions of the motor-bottleneck hypothesis than those of the response-monitoring hypothesis, which are summarized in Table 1. First, the Task-2 difficulty effect on RT2 was reduced with a Task-1 motor response. This suggests that the response-related bottleneck delays the motor stage of Task 2, as asserted by the motor-bottleneck hypothesis, rather than delaying the response-selection stage of Task 2 as asserted by the response-monitoring hypothesis. In addition, there was a three-way interaction across the three experimental factors, which is more consistent with the motor-bottleneck hypothesis, although the nature of the interaction was not exactly what the motor-bottleneck hypothesis predicted, as discussed below. Furthermore, the relatively consistent premotoric duration of Task 2 (as measured by S2-LRP) regardless of Task-1 motor requirement supports the motor-bottleneck hypothesis, which places the locus of the response-related bottleneck after the response-selection stage of Task 2; in contrast, the response-monitoring hypothesis predicts a systematic lengthening of the premotoric duration when Task 1 requires a motor response. However, we should note that the last prediction of the motor-bottleneck hypothesis—selective lengthening of LRP-R2 in the easy condition of Task 2 with Task-1 motor response at the short SOA—was not supported.

Although the overall results were more consistent with the motor-bottleneck hypothesis, there were some effects that were not predicted by either hypothesis, especially at the long SOA. For example, as reported earlier, at the long SOA, the comparison between Go trials and NoGo trials showed that the difficulty effect was significantly smaller in the Go trials (231 ms) than in the NoGo trials (304 ms). Neither hypothesis predicts such a reduced Task-2 difficulty effect due to Task-1 motor response at the long SOA. As another example of unexpected results at the long SOA, there was a significant delay of RT2 (by 62 ms) in the Go trials relative to NoGo trials—response-related interference even at the long SOA—*F*(1, 25) = 5.641, *p* = 0.026, *η*_*p*_^2^ = 0.184. However, again, neither hypothesis assumed that Task 2 would be modulated by Task-1 motor requirement at the long SOA.

We speculate that these unexpected results at the long SOA were due to residual processing overlap between the two tasks even in the long-SOA condition employed in Experiment 1. Specifically, although the SOA of 900 ms corresponds to the typical long-SOA condition in PRP studies, it was not sufficiently long in Experiment 1 considering that the mean RT1 in the Go trials was 1179 ms. Therefore, even in the long-SOA condition, Task 1 was often not completed, yielding the unexpected results (i.e., reduced Task-2 difficulty effect due to Task-1 motor response as well as the response-related interference). This speculation motivated us to replicate Experiment 1 with an even longer long-SOA condition in Experiment 2.

## Experiment 2

Experiment 2 replicated Experiment 1 with a long SOA of 1200 ms rather than 900 ms. With this longer SOA, we expected that Task 1 would show even less influence on Task 2 at the long SOA than it did in Experiment 1. Therefore, the overall effect of SOA on Task 2 should be greater than the one observed in Experiment 1 (i.e., a greater PRP effect). Similarly, we also expected that Task-1 motor requirement would show diminished influence on Task 2 relative to Experiment 1 primarily due to the lack of its effect at the new long-SOA condition. However, there should still be an observable effect of Task-1 motor requirement on Task 2 at the short SOA.

### Method

#### Participants

Fifteen undergraduate students from Kennesaw State University participated for partial course credit. One participant was excluded because of low Task-1 accuracy (lower than 80%). Among the remaining participants (12 women, two men, *M*_age_ = 21, age range = 18–33), two participants were African American, 11 were White (non-Hispanic), and one was Asian. Two reported being left-handed and all reported normal or corrected-to-normal vision.

#### Stimuli and procedure

Experiment 2 was identical to Experiment 1 except that the long SOA changed from 900 to 1200 ms.

## Results

### RT analysis

Data were handled in the same manner as in Experiment 1. Regarding RT1, SOA and Task-2 difficulty did not have a main effect (*p*s > 0.05), although their interaction was significant, *F*(1, 13) = 11.808, *p* = 0.004, *η*_*p*_^2^ = 0.476. Specifically, the effect of Task-2 difficulty on RT1 was reversed at the long SOA (− 23 ms) compared to the short SOA (37 ms).

There was a significant PRP effect—RT2 was greater at the short SOA (1350 ms) than at the long SOA (703 ms) by 647 ms, *F*(1, 13) = 1010, *p* < 0.001, *η*_*p*_^2^ = 0.987 (see Table 2 and lower panels in Fig. 5). As we had expected, the current PRP effect was greater than the one in Experiment 1 (505 ms), *F*(1, 38) = 17.983, *p* < 0.001, *η*_*p*_^2^ = 0.321, suggesting that the new long-SOA condition further separated the two tasks than the corresponding condition did in Experiment 1, minimizing the interferences between tasks at the long SOA.

Regarding the effect of Task-1 motor requirement, RT2 in the Go trials (1042 ms) was longer than RT2 in the NoGo trials (1011 ms) by 31 ms, which was marginally significant, *F*(1, 13) = 4.160, *p* = 0.062, *η*_*p*_^2^ = 0.242. As expected, in Experiment 2, the effect of Task-1 motor requirement on RT2 was smaller than the corresponding effect in Experiment 1 (86 ms)—it was less than half of the effect in Experiment 1, which was a marginally significant reduction, *F*(1, 38) = 3.593, *p* = 0.066, *η*_*p*_^2^ = 0.086. However, there was still a significant effect of Task-1 motor requirement on RT2 at the short SOA (69 ms), *F*(1, 13) = 13.975, *p* = 0.002, *η*_*p*_^2^ = 0.518, which disappeared at the long SOA (-7 ms), *F*(1, 13) = 0.158, *p* = 697, *η*_*p*_^2^ = 0.012. Therefore, the new long-SOA condition seemed to reduce the response-related interference at the long SOA. Notably, as in Experiment 1, the overall delay of RT2 at the short SOA relative to the long SOA—the PRP effect—was more prominent in the Go trials (685 ms) than in the NoGo trials (610 ms), indicating that when Task 1 requires a motor response, the PRP effect is even greater, supporting the contribution of the response-related interference in the typical PRP effect involving Task-1 motor response.

The Task-2 difficulty manipulation lengthened RT2 in the difficult condition (834 ms) than in the easy condition (572 ms) by 262 ms at the long SOA, *F*(1, 13) = 57.616, *p* < 0.001, *η*_*p*_^2^ = 0.816, which was similar to the corresponding effect in Experiment 1 (268 ms), *F*(1, 38) = 0.019, *p* = 0.890, *η*_*p*_^2^ = 0.001. Again, the primary focus of the current study is whether this Task-2 difficulty effect is reduced at the short SOA due to Task-1 motor response and whether there is a three-way interaction across the three experimental factors (the two-way interaction between Task-1 motor requirement and Task-2 difficulty appears at the short SOA but not at the long SOA). Looking at the short SOA only, as in Experiment 1, there was a significant reduction of Task-2 difficulty effect due to Task-1 motor response, *F*(1, 13) = 9.199, *p* = 0.010, *η*_*p*_^2^ = 0.414: Task-2 difficulty effect was 167 ms in the Go trials while it was 286 ms in the NoGo trials (119 ms reduction), indicating absorption of the Task-2 difficulty effect by the response-related bottleneck, consistent with the motor-bottleneck hypothesis. Somewhat unexpectedly, looking at the long SOA only, there was still a significant reduction of the difficulty effect in the Go trials (233 ms) than in the NoGo trials (291 ms)—58 ms reduction, suggesting a residual response-related interference even in the new long-SOA condition in Experiment 2. Finally, there was a marginally significant three-way interaction across Task-1 motor requirement, Task-2 difficulty, and SOA, *F*(1, 13) = 2.782, *p* = 0.119, *η*_*p*_^2^ = 0.176 (see lower panels in Fig. 5). Notably, consistent with the motor-bottleneck hypothesis, in the NoGo trials, the difficulty effect was consistent across SOAs (286 ms at the short SOA vs. 291 ms at the long SOA), *F*(1, 13) = 0.057, *p* = 0.816, *η*_*p*_^2^ = 0.004; however, in the Go trials, the difficulty effect was significantly reduced at the short SOA than at the long SOA (167 ms at the short SOA vs. 233 ms at the long SOA), *F*(1, 13) = 9.686, *p* = 0.008, *η*_*p*_^2^ = 0.427. Such a prominent reduction in the Task-2 difficulty effect due to the Task-1 motor response at the short SOA relative to the long SOA is the most distinguishable prediction of the motor-bottleneck hypothesis.

### ACC analysis

ACC1 was significantly lower at the short SOA than at the long SOA (94% vs. 97%), *F*(1, 13) = 28.704, *p* < 0.001, *η*_*p*_^2^ = 0.688. Participants performed significantly better when Task 2 was difficult (96%) than when it was easy (94%), *F*(1, 13) = 7.126, *p* = 0.019, *η*_*p*_^2^ = 0.354. Such an effect of Task-2 difficulty on ACC1 was even greater at the short SOA (− 4%) than at the long SOA (0%), *F*(1, 13) = 4.735, *p* = 0.049, *η*_*p*_^2^ = 0.267. There was a significant three-way interaction across Task-2 difficulty, SOA, and Task-1 motor requirement, suggesting that the greater effect of Task-2 difficulty on ACC1 at the short SOA than at the long SOA was even greater in the Go trials (− 3%) than in the NoGo trials (− 1%), *F*(1, 13) = 5.981, *p* = 0.029, *η*_*p*_^2^ = 0.315.

ACC2 was significantly lower at the short SOA than at the long SOA (92% vs. 96%), *F*(1, 13) = 32.250, *p* < 0.001, *η*_*p*_^2^ = 0.713. Task-2 difficulty manipulation showed the expected effect on ACC2: 96% vs. 92% in the easy and difficult conditions, *F*(1, 13) = 6.765, *p* < 0.022, *η*_*p*_^2^ = 0.342. There was a significant interaction between Task-2 difficulty and SOA, *F*(1, 13) = 26.664, *p* < 0.001, *η*_*p*_^2^ = 0.672, suggesting that the difficulty effect was smaller at the short SOA (1%) than at the long SOA (7%). However, Task-1 motor requirement did not show any significant main or interaction effects on ACC2 (*p*s > 0.05).

### LRP analysis

LRP was calculated and analyzed in the same manner as in Experiment 1. The only significant effect on S2-LRP onset was the main effect of SOA (*p* = 0.019): the onset of the S2-LRP was significantly delayed at the short SOA (1019 ms) relative to the long SOA (367 ms), indicating a lengthening of the premotoric duration of Task 2 as task overlap increased, consistent with the response-selection bottleneck hypothesis. No other effects or interactions on S2-LPR were significant (*p* > 0.05). Notably, as in Experiment 1, unlike what the response-monitoring hypothesis predicted (see also Table 1), there was not a significant main effect of Task-1 motor requirement on S2-LRP onset at the short SOA.

Regarding LRP-R2 onset, the Task-2 difficulty manipulation showed a marginally significant trend on LRP-R2 onset (*p* = 0.081): LRP-R2 occurred earlier in the easy condition (− 132 ms) than in the difficult condition (− 108 ms). However, unlike what the motor-bottleneck hypothesis predicted—a selective lengthening of LRP-R2 when Task 1 requires a motor response in the easy condition of Task 2 at the short SOA—there was no statistically significant evidence to support this prediction.

### Discussion

Relative to Experiment 1, the new long-SOA condition in Experiment 2 (1200 ms rather than 900 ms) yielded a greater PRP effect combined with a smaller effect of Task-1 motor requirement at the long SOA. Most notably, as in Experiment 1 and consistent with the motor-bottleneck hypothesis, there was a significant reduction in the Task-2 difficulty effect when Task 1 required motor response, which was selectively prominent at the short SOA; this pattern was supported by a significant two-way interaction between Task-1 motor requirement and Task-2 difficulty on RT2 at the short SOA as well as a marginally significant three-way interaction. In addition, as in Experiment 1 and again consistent with the motor-bottleneck hypothesis, the S2-LRP was consistent regardless of the Task-1 motor requirement. Such a result is difficult to reconcile with the response-monitoring hypotheses, which predicted a significant main effect of Task-1 motor requirement on the S2-LRP. However, one of the predictions of the motor-bottleneck hypothesis—selective lengthening of LRP-R2 in the easy condition of Task 2 due to Task-1 motor response—was not supported.

One virtue of the standard PRP paradigm, compared to paradigms in which single-task trials and dual-task trials are run in separate blocks, is that the pre-trial preparatory state is identical for long SOAs and short SOAs. Nevertheless, it is possible for participants to become increasingly more prepared for Task 2 at long SOAs, once Task 1 has been responded to. In addition, the temporal onset of the Task-2 stimulus is potentially more predictable at long SOAs. This extra preparation would be even more likely in Experiment 2, which employed an even longer long-SOA (1200 ms) than did Experiment 1 (long SOA of 900 ms).

Could this extra preparation at long SOAs explain the key interactions on RT2 in the present experiment? The extra-preparation account is consistent with the relatively short RT2 in the long-SOA condition of Experiment 2 than in Experiment 1 (703 ms vs. 875 ms), though that RT difference might also reflect a reduced likelihood of encountering a bottleneck. According to Müller-Gethmann, Ulrich, and Rinkenauer (2003), increased preparation might very well facilitate the pre-motoric stages of Task 2. Importantly, however, the increased preparedness for S2 in the long-SOA condition should reduce the difficulty effect. Yet, that is the exact opposite of what we found (i.e., the difficulty effect was greater at the long SOA than the short SOA). Therefore, although extra preparation at long SOAs is a strong possibility, it cannot explain the observed interaction between SOA and Task-2 difficulty.

## General discussion

Regarding the cause of dual-task interference, researchers traditionally have considered response selection as the sole source, or at least the primary source, while ignoring possible contributions from motor responses (e.g., Pashler, 1984, 1994; Tombu & Jolicœur, 2003). However, we observed a greater delay in RT2 when Task 1 required a motor response than when it did not (by 86 ms in Experiment 1 and by 31 ms in Experiment 2). This delay was even more prominent at the short SOA: a 110-ms delay in Experiment 1 and 69-ms delay in Experiment 2. We observed such a delay of RT2 due to the Task-1 motor response even though our sample was young and our responses—button presses—were very simple, familiar, fast, and ballistic. The present results add to the evidence that dual-task costs are not due exclusively to response-selection interference. Instead, motoric processes also contribute to dual-task costs as some previous researchers have suggested (Bratzke et al., 2008; De Jong, 1993; Keele, 1973; Ulrich et al., 2006).

The present data not only suggest response-related interference but also shed light on the nature of that interference. Two competing views have been investigated. The motor-bottleneck hypothesis asserts that the Task-1 motor response temporarily delays the motor stage of Task 2 (Keele, 1973); in contrast, the response-monitoring hypothesis asserts that Task-1 motor response is accompanied by a resource-demanding monitoring process that further delays the response-selection stage of Task 2 (Welford, 1952). Previous studies reported inconsistent conclusions regarding the two hypotheses. Behavioral data obtained with a Task-1 motor-response manipulation supported the motor-bottleneck hypothesis (Bratzke et al., 2008, 2009), while electrophysiological data with a focus on residual PRP effect supported the response-monitoring hypothesis (Jentzsch et al., 2007). The aim of the present study was to test the two hypotheses by designing a traditional PRP experiment for which the two hypotheses predict different behavioral and electrophysiological results. Specifically, we manipulated the Task-1 motor requirement, Task-2 response-selection difficulty, and SOA in two experiments.

According to the motor-bottleneck hypothesis, the Task-2 difficulty effect can be absorbed into the response-related bottleneck that occurs after the response-selection stage but before the motor stage of Task 2. At the short SOA in both experiments, we observed such a reduction in the Task-2 difficulty effect when Task 1 required a motor response. The response-monitoring hypothesis, however, does not allow such a reduction because it asserts that the response-related bottleneck occurs before the response-selection stage of Task 2. Moreover, although the response-monitoring hypothesis predicted a significant lengthening of the premotoric duration of Task 2 when Task-1 required a motor response, this effect was not statistically significant; furthermore, in Experiment 1, the numerical trend also behaved in the opposite direction to what was expected by the hypothesis. However, one of the predictions of the motor-bottleneck hypothesis, regarding the lengthening of the LRP-R2 for the easy condition on Go trials, was not supported. We should note that the LRP results, which were not predicted correctly by either hypothesis, might reflect a mistaken assumption about when the LRP (reflecting motor preparation) begins. For example, we assumed that motor preparation begins following selection of the Task-1 response, but it might instead need to wait until the motor refractory period has ended.

In any case, the data are, in general, much more consistent with the motor-bottleneck hypothesis, which asserts that the nature of the response-related bottleneck is motoric: The Task-1 motor stage temporarily delays the motor stage of Task 2. Therefore, the widely accepted explanation of the dual-task interference in terms of a pure response-selection bottleneck, as depicted in Fig. 1 (Pashler, 1984, 1994), may need to be modified by adding an additional period of slack, which is smaller than the cognitive slack, between the response-selection stage and motor stage of Task 2.

### But cannot we move limbs simultaneously, without a delay?

Response-related interference could play an important role of protecting the organism from making two conflicting responses. For example, when a person wants to cut vegetables with a knife and also check a watch on the wrist, the response-related interference may serve to space out the two possibly conflicting motor responses. However, as we know from watching people dancing at a party, people appear to move their limbs simultaneously and smoothly. These anecdotal observations seemingly go against the notion of response-related interference in dual- or multi-tasking, so why is response-related interference observed in a laboratory? There are a few viable explanations. First, the duration of the delay due to response-related interference is somewhat short—in this study, it was less than 100 ms. Such a short delay may not be obvious to the performers or watchers. In addition, extensive practice with dual-tasking may allow the performers, especially experts, to eventually bypass the motoric bottleneck. For example, they might be able to chunk multiple responses together and jointly implement them as a single motor process (for evidence of bypassing the central bottleneck with practice, or at least shortening it dramatically, see Hazeltine, Ruthruff, & Remington, 2006; Maquestiaux, Laguë-Beauvais, Ruthruff, & Bherer, 2008; Ruthruff, Van Selst, Johnston, & Remington, 2006; note, however, that Ruthruff et al. did not find efficient dual-tasking between two tasks with manual responses, as in the present study).

### Implications for PRP studies using locus-of-slack logic

Numerous studies have utilized locus-of-slack logic (McCann & Johnston, 1992; Pashler & Johnston, 1989) to determine whether a certain mental process is “automatic” in the sense that it can operate without central attentional resources. Locus-of-slack logic requires manipulating not only the SOA but also the difficulty of the target mental process embedded in Task 2. For example, Jung, Ruthruff, Tybur, Gaspelin, and Miller (2012) varied the difficulty of an attractiveness judgment (Task 2) by presenting faces that were either close to or far from the dividing line between attractive and unattractive. If the difficulty effect interacts underadditively with SOA, then such results indicate that the target mental process has occurred prior to the response selection, especially during the cognitive slack where the attentional resources are not available, and therefore the target process is automatic; but if it interacts additively with SOA, then it does require the resources (i.e., non-automatic, as reported by Jung et al., 2012).

The present findings have implications for PRP studies using the locus-of-slack logic. In those studies, participants are typically required to respond to both tasks. Therefore, based on the present findings, Task-2 performance might be limited by both a response-selection bottleneck and a motoric bottleneck. Although researchers have traditionally taken underadditive interactions to indicate the automaticity of the target mental process in Task 2, the present study offers an alternative explanation. The target process might have occurred *after* the response-selection bottleneck (i.e., only after central attentional resources became available), but the difficulty manipulation on the target process was absorbed into the slack created by the motoric bottleneck.

As a remedy for this issue, researchers who utilize the locus-of-slack logic may consider adopting a go–no-go Task 1. If a target process is truly automatic—i.e., does not require the central attentional resources responsible for the response-selection bottleneck—then the underadditivity between difficulty and SOA should be observed for NoGo trials as well as Go trials. Or researchers could adopt a Task 2 with a large (greater than 100 ms) difficulty effect so that the effect cannot be completely absorbed into the relatively short motoric-bottleneck delay. If underadditivity is found using locus-of-slack logic, converging approaches are needed to confirm the findings.

We should also note that the present study does not pose a problem for locus-of-slack logic for cases in which additivity was found. However, it does raise a further problem: true additivity should be very difficult to obtain if there really is a late motoric bottleneck. One possibility is that researchers could have ignored a trend towards underadditivity. For example, in two experiments, Jung, White, and Powanda (2019) tested the automaticity of gender categorization using locus-of-slack logic. In each experiment, they did not observe strong underadditivity. However, when they combined the data from both experiments, there was a statistically significant trend of underadditivity. That is, due to a relatively low power of an individual experiment, researchers could have ignored an underadditive trend. Another possibility is that when Task 1 involves vocal responses rather than manual responses (e.g., McCann & Johnston, 1992; Van Selst, Ruthruff, & Johnston, 1999), the motoric bottleneck may be diminished or absent, yielding true additivity.

## Footnotes

1 Source code and the datasets generated and analyzed during the current study are available in the following repository http://facultyweb.kennesaw.edu/kjung2/research.php.
